# The Effects of *Perilla frutescens* Extracts on IgA Nephropathy: A Systematic Review and Meta-Analysis

**DOI:** 10.3390/ph16070988

**Published:** 2023-07-10

**Authors:** Gigi Adam, Ana-Maria Adam, Silvia Robu, Valeriu Harabor, Anamaria Harabor, Aurel Nechita, Denisa Batir Marin, Ionela-Daniela Morariu, Oana Cioanca, Ingrid-Andrada Vasilache, Monica Hancianu

**Affiliations:** 1 Faculty of Pharmacy, “Grigore T. Popa” University of Medicine and Pharmacy, 16 Universitatii Street, 700115 Iasi, Romania; adam.gigi20@gmail.com (G.A.);; 2 Department of Pharmaceutical Sciences, Faculty of Medicine and Pharmacy, ‘Dunarea de Jos’ University, 800216 Galati, Romania; 3 Clinical and Surgical Department, Faculty of Medicine and Pharmacy, ‘Dunarea de Jos’ University, 800216 Galati, Romaniaharaboranamaria@yahoo.com (A.H.);; 4 Department of Obstetrics and Gynecology, ‘Grigore T. Popa’ University of Medicine and Pharmacy, 700115 Iasi, Romania

**Keywords:** *Perilla frutescens*, IgA nephropathy, mesangioproliferative glomerulonephritis, extract

## Abstract

(1) Background: Chronic renal disorders (CRD) are associated with significant comorbidities and necessitate complex therapeutic management. As time passed, *Perilla frutescens* (PF) became a promising therapeutic option for CRD. The aim of this systematic review and meta-analysis was to outline the therapeutic effects of PF extracts on various models of immunoglobulin a (IgA) nephropathy; (2) Methods: Medline, Embase, and Cochrane databases were used to find relevant studies. All prospective interventional studies that evaluated the effect of PF extract versus placebo on rat models of chronic renal disorders were assessed according to the international guidelines; (3) Results: Our search yielded 23 unique records, out of which only five were included in the analysis. Our results showed that administration of PF extracts led to a statistically significant reduction in proteinuria and PCNA levels in rats that received high doses of the extract as well as in the PCNA level and DNA synthesis in rats that received low doses of the extract. The evaluated outcomes benefited from a low degree of heterogeneity; (4) Conclusions: Some of the evaluated outcomes were significantly reduced by both high and low doses of extracts from *Perilla frutescens*. Further studies are needed to determine the exact effect over IgA nephropathy in human subjects.

## 1. Introduction

Chronic kidney disease (CKD) is a major public health problem that can drain significant financial and social resources. Furthermore, it is a risk factor for hypertension and cardiovascular disease, both of which are significant causes of morbidity and mortality in the general population of most countries [1,2]. The global prevalence of CKD (stages 1–5) is estimated to be 3–18%, with a greater prevalence in females than males, at least among those aged >40 years at the time of CKD diagnosis, and a significant contribution from the geriatric population [3,4,5,6]. According to a systematic analysis by Mills et al., the age-standardized prevalence of CKD stages 1–5 among individuals living in 32 countries was 10.4% among men and 11.8% among women (range 4.5–25.7% for men and 4.1–18.4% for women) [7].

Renal disorders comprise a diversity of pathological entities aside from CKD, including nephropathies, glomerulopathies, acute kidney injury, autoimmune disorders, and tumors [8]. Mesangioproliferative glomerulonephritis (MesPGN) is a distinct glomerular response pattern characterized by a diffuse or focal increase in the number of mesangial cells and expansion of the extracellular matrix in the glomerular mesangium, with or without immunoglobulin or complement deposition [9]. This pattern can be encountered in several groups of renal diseases including IgA nephropathy (IgAN), IgM nephropathy (IgMN), lupus nephritis (LN), C1q nephropathy (C1qN), and other entities [10,11,12].

IgAN is the most frequent type of primary glomerulonephritis and one of the leading causes of renal failure [13]. The prevalence of IgAN differs significantly depending on racial background, with East Asians having the highest incidence, followed by Caucasians [14]. Its incidence has been estimated to be 2–10 per 100,000 person-years, with peaks occurring throughout the second and third decades of life [15].

The diagnosis of IgAN is primarily conducted using histopathological examination of renal biopsies, and the current treatment involves maintaining appropriate blood pressure control with an angiotensin converting enzyme (ACE) inhibitor or angiotensin receptor blocker (ARB) medication, corticosteroid therapy, immunosuppression, and ultimately kidney transplant [16]. The individualized management of these disorders is often difficult due to their great complexity, and numerous herbal extracts have been investigated as potential therapeutic means [17,18,19,20].

*Perilla frutescens* (L.) Britt., a member of the Labiatae family, is widely spread around the world, particularly in Eastern Asian countries [21,22]. Flavonoids, volatile oils, fatty acids, triterpenes, phenolic compounds, and other substances have been extracted and identified from this plant [22]. Moreover, a recent review highlighted substantial therapeutic properties of this herb, including antioxidant, antibacterial, anti-allergic, antidepressant, anti-inflammatory, antitumoral, and neuroprotection actions [23].

Although these effects resulted from the analysis of in vitro and in vivo studies, there were few clinical trials that outlined significant therapeutic effects for human subjects. Attenuation of age-related cognitive decline, antiallergic, antioxidant, prokinetic, and antispasmodic effects are among the proven therapeutic proprieties of *Perilla frutescens* in these studies [24,25,26,27,28,29].

A recent literature review outlined the main applications of *Perilla frutescens* in clinical practice using data derived from human studies and indicated that the extracts from this herb have demonstrated anti-inflammatory, antiallergic, antioxidant, and hypolipemiant effects in humans [30]. Other miscellaneous effects referred to its antibacterial and antifungal proprieties as well as its role as a prebiotic and reliever of gastrointestinal discomfort.

MesPGN is a renal disorder with an important inflammatory component, and it constitutes an interesting target for possible therapeutic approaches based on *Perilla frutescens* extracts. The effects of *Perilla frutescens* extracts are poorly studied in the literature, and the aim of this systematic review and meta-analysis is to outline their therapeutic effects on IgA nephropathy.

## 2. Results

**Figure 1:**
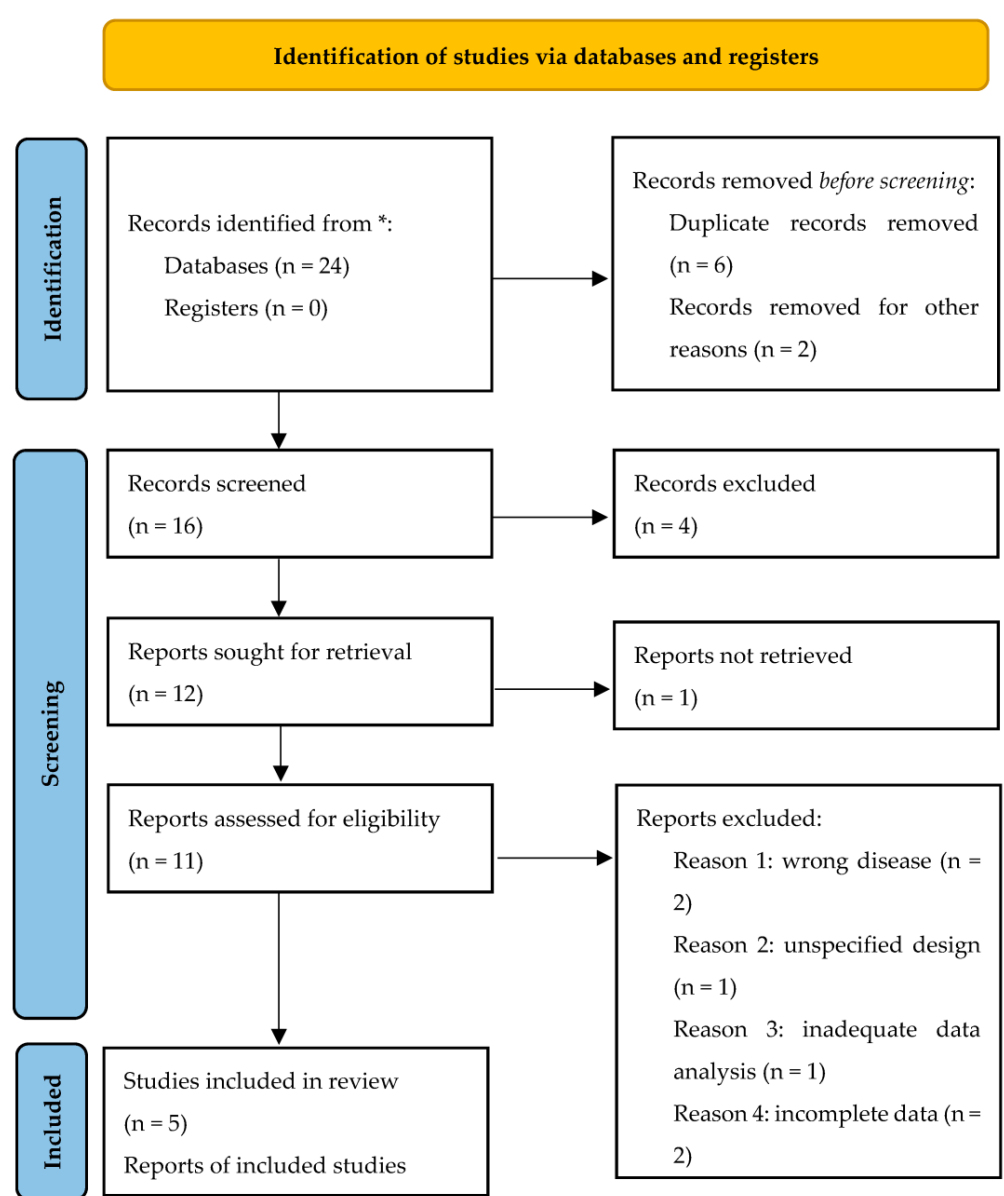
Flow diagram of search and selection of studies.

Our search yielded 23 unique records, out of which only five were included in our systematic review after abstract and full text screening (Figure 1). We did not retrieve additional items after screening references and related articles.

The characteristics of the included studies are presented in Table 1. A total of 158 rat models and five studies [22,23,24,25,26] were included in the meta-analysis, while other studies were excluded mainly because the statistical analyses were not informative when using insufficient data. For the purpose of this meta-analysis, we evaluated the therapeutic effects of *Perilla frutescens* on animal models that were mentioned at least twice.

The quality of the included studies was assessed using SYRCLE’s risk of bias tool for animal studies (Table 2). Only one study randomly allocated animal models to the intervention and control groups [35]. It is unclear if the other studies randomly allocated animal subjects to a specific intervention. All studies evaluated similar animal models, but the risk was unclear regarding the concealment of different group segregations by investigators.

The housing of these animals was not extensively described in all evaluated studies; thus, the lack of details resulted in an unclear risk of bias assessment regarding this domain. No study found a high risk of bias in one domain (subject selection). The investigators were not blinded from knowledge of which intervention each animal received during the experiment in all studies performed by Makino et colleagues, while this aspect was unclear for the study performed by Sakurai et al. [35]. These aspects suggest a possible performance bias of the evaluated studies.

None of the studies indicated that the animals were selected at random for outcome assessment, and it remains unclear if the outcome assessor was blinded, thus suggesting a possible risk of detection bias.

All studies adequately addressed incomplete outcome data, and the reports of the studies were free of selective outcome reporting, thus resulting in a lack of attrition or reporting bias. No other biases were identified while using the SYRCLE’s risk of bias tool. No studies were excluded from the analysis based on their quality.

**Figure 2:**
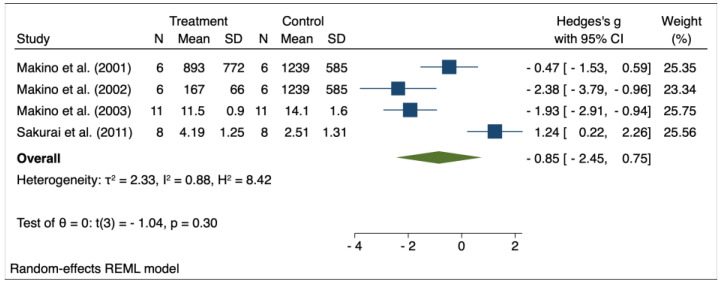
Forrest plot for overall effect size of low-dose Perilla frutescens versus placebo on proteinuria [32,33,34,35].

**Figure 3:**
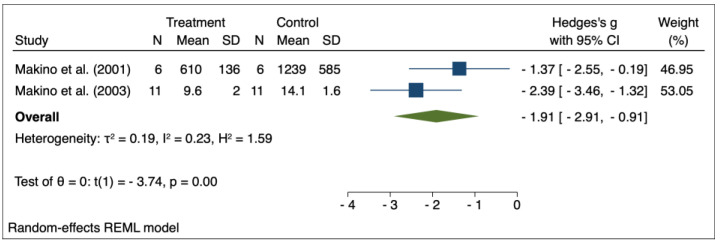
Forrest plot for overall effect size of high-dose Perilla frutescens versus placebo on proteinuria [32,34].

**Figure 4:**
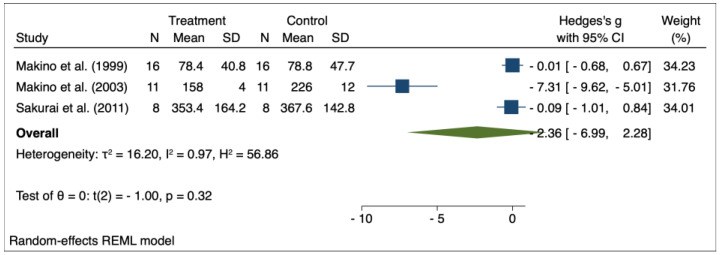
Forrest plot for overall effect size of low-dose Perilla frutescens versus placebo on serum levels of immunoglobulin A [31,34,35].

**Figure 5:**
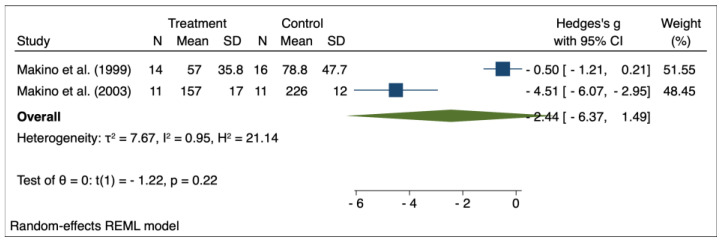
Forrest plot for overall effect size of high-dose Perilla frutescens versus placebo on serum levels of immunoglobulin A [31,34].

**Figure 6:**
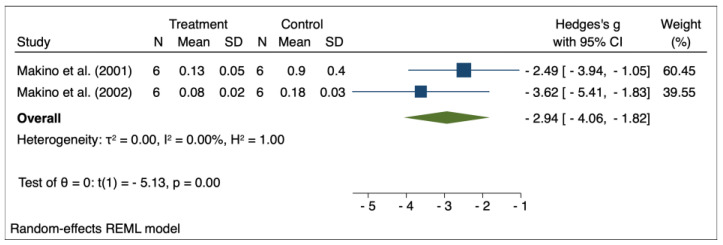
Forrest plot for overall effect size of low-dose *Perilla frutescens* versus placebo on the positivity for proliferating cell nuclear antigen (PCNA) [32,33].

**Figure 7:**
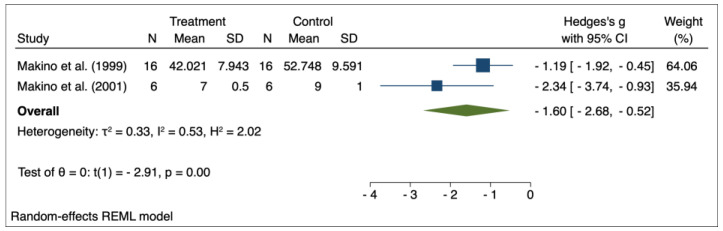
Forrest plot for overall effect size of low-dose *Perilla frutescens* versus placebo on the DNA synthesis [31,32].

**Figure 8:**
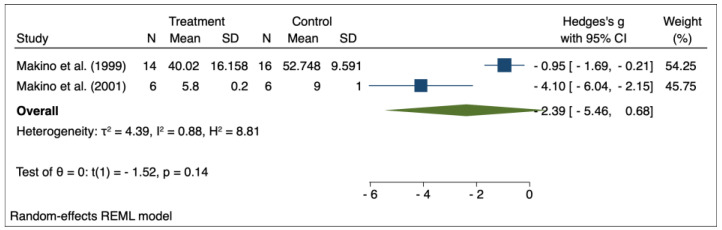
Forrest plot for overall effect size of high-dose *Perilla frutescens* versus placebo on the DNA synthesis [31,32].

The results of the random effects model used for meta-analysis are presented in Table 3, and the forest plots are depicted in Figure 2, Figure 3, Figure 4, Figure 5, Figure 6, Figure 7 and Figure 8. Administration of *Perilla frutescens* extracts determined a statistically significant reduction in the proteinuria level in rats that received high doses of the herb (*p* < 0.001), in the PCNA level for both groups versus placebo (*p* < 0.001), as well as in the [^3^H] thymidine incorporation as a measure of DNA synthesis for the low-dose group (*p* < 0.001). On the other hand, we could determine a great deal of heterogeneity between studies, ranging from 88–100% for the majority of the reported outcomes. A low to medium heterogeneity (I^2^: 0–53%) was observed for studies reporting the following outcomes: proteinuria for the high-dose versus placebo groups, and PCNA or DNA synthesis for the low-dose versus placebo groups.

**Figure 9:**
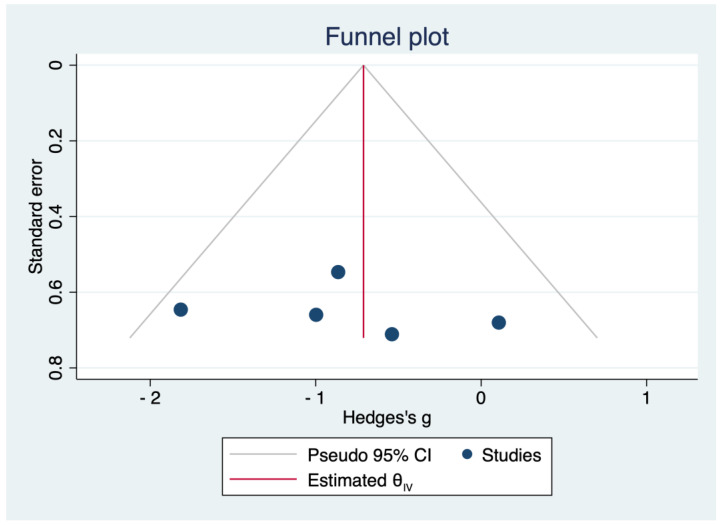
Funnel plot for publication bias.

The funnel plot of the pooled treatment effects estimated from individual studies against a measure of study size is presented in Figure 9. Its shape is slightly asymmetrical, indicating a possible publication bias, and there are small studies of inadequate quality whose results are biased towards larger beneficial effects.

## 3. Discussion

This systematic review and meta-analysis evaluated the therapeutic effects of *Perilla frutescens* on various animal models of IgA nephropathy. Our results showed that administration of *Perilla frutescens* extracts led to a statistically significant reduction in proteinuria and PCNA levels in rats that received high doses of the extract as well as in the PCNA level and DNA synthesis in rats that received low doses of the extract. The evaluated outcomes benefited from a low degree of heterogeneity.

Proteinuria was identified as the most widely recognized and well-studied risk factor for progression to end-stage chronic renal disease in IgAN [36]. It can be used as a basis for accelerated approval of therapies intended to treat serious or life-threatening conditions, such as IgAN.

Numerous observational studies have established the significance of both the extent and duration of proteinuria in relation to the progression of IgAN [36,37,38]. The degree of advancement is notably slower in individuals who excrete less than 1 g/day, while it is most pronounced in those who excrete more than 3 to 3.5 g/day.

In a study that evaluated 542 adult patients diagnosed with IgAN over a period of 6.5 years, it was found that the rate of decline in kidney function was significantly higher in patients with consistent proteinuria levels exceeding 3 g/day compared to those with protein excretion levels below 1 g/day [39]. The former group experienced a decline of 0.72 mL/min/1.73 m^2^ per month, which was 24 times faster than the latter group’s decline rate of 0.04 mL/min/1.73 m^2^ per month. This study highlighted the negative impact of sustained proteinuria on kidney function in patients with IgAN.

Patients exhibiting proteinuria levels exceeding 3 g/day who achieved partial remission (less than 1 g/day) demonstrated a comparable rate of progression to renal failure as those who sustained proteinuria levels below 1 g/day from the time of presentation [39]. The correlation between elevated proteinuria levels and a poorer prognosis can be attributed, at least partially, to proteinuria serving as an indicator of the gravity of the glomerular ailment.

Several studies indicate that nearly one-third of patients exhibiting high-risk histologic features, particularly those with notable hematuria, display proteinuria levels below 1 g/day and subsequently encounter a decline in kidney function [40]. The presence of proteinuria below 1 g/day as an isolated clinical finding cannot ensure a favorable prognosis or imply the absence of a necessity for intervention.

[H^3^] thymidine incorporation was used in multiple rat models and human cell lines as an indirect marker of DNA synthesis and cell proliferation [41,42,43,44]. Thus, a reduction in [H^3^] thymidine incorporation translates into a reduction in cell proliferation, in our case of mesangial proliferation.

Recent research has produced a four-hit concept for the development and/or clinical manifestations of IgA nephropathy [45]. Galactose shortage of some O-linked glycans in the hinge region of IgA1 initiates a chain of events that may result in renal damage. IgA1 galactose-deficient O-glycans are made up of terminal N-acetylgalactosamine (GalNAc) or sialylated GalNAc [46,47]. Anti-glycan antibodies identify the hinge-region glycans of IgA1 with terminal Gal-NAc [46,48], resulting in the formation of nephrotoxic circulating immune complexes that deposit in the glomerular mesangium, causing kidney damage [46]. Numerous literature reviews have outlined the association between high serum IgA levels and IgAN severity and activity [49,50].

Proliferating cell nuclear antigen (PCNA) is an acidic nuclear protein that increases from the late G1 to S phases of the cell cycle and whose detection parallels other standard methods for assessing cell proliferation [51,52]. Nakopoulou et al. investigated the PCNA expression in normal and diseased human kidneys and defined a possible correlation of its expression with various types of glomerulonephritis (GN) [53]. The authors demonstrated a significantly higher expression on the histopathological probes from subjects with IgA nephropathy.

While clinical features have been identified as robust prognostic indicators, specific histological markers identified during kidney biopsy in IgAN patients have been linked to an elevated risk of disease progression. These markers comprise both indicators of marked inflammatory disease, such as crescent formation and immune deposits in the capillary loops, alongside the mesangial deposits that are universally present in all patients [54,55]. Additionally, they encompass markers of persistent fibrotic disease, such as glomerulosclerosis, tubular atrophy, interstitial fibrosis, and vascular disease, as documented in various sources [56,57,58]. The incorporation of contemporary pathological discoveries enhances the precision of prognostication beyond the exclusive consideration of clinical characteristics upon evaluation during the kidney biopsy.

The utilization of the revised Oxford classification of IgAN involves the evaluation of kidney biopsies through the assessment of five histologic variables, namely mesangial hypercellularity (M), endocapillary hypercellularity (E), segmental glomerulosclerosis (S), tubular atrophy/interstitial fibrosis (T), and crescents (C) [59].

Empirical evidence has demonstrated that each of the aforementioned variables possesses the ability to predict kidney outcome in isolation, irrespective of clinical data. Moreover, a recent international multiethnic cohort study on 3927 patients evaluated the predictive performance of a model that comprised clinical and histological risk factors for the prediction of the risk of a 50% decline in kidney function or end-stage renal disease in patients with IgA nephropathy [60]. The model was internally and externally validated, obtaining good predictive results. As such, the influence of novel therapeutic agents over these markers could be assessed in clinical trials that could potentially establish various correlations between treatment effects and degree of positivity as well as with clinical outcomes.

*Perilla frutescens* contains a high amount of n–3 polyunsaturated fatty acids (PUFA) that have been proven to modulate immune and inflammatory responses [61,62]. PUFA has been observed to exert beneficial effects on various stages of renal fibrosis, including mesangial cell activation and proliferation as well as extracellular matrix protein synthesis. These effects are mediated through the regulation of pro-inflammatory cytokine production, renin and nitric oxide (NO) systems, and the expression of peroxisome proliferator-activated receptor genes [63]. As we previously outlined, IgA nephropathy is a kidney disorder with an important inflammatory component; thus, we hypothesize that *Perilla frutescens* extracts could potentially have an impact on this disease progression. However, there are no studies on human subjects that have analyzed this topic.

These arguments support the hypothesis that the evaluated outcomes were significantly reduced by both high and low doses of extracts from *Perilla frutescens*. Thus, they could be further evaluated in a clinical trial setting, on a larger cohort of patients, in order to determine the exact effect on chronic renal disorders and their impact on clinical and histological markers of disease progression.

Mesangioproliferative glomerulonephritis has an important inflammatory pathophysiological background that comprises two main elements: deposition of immune complexes or monoclonal immunoglobulins leading to activation of complement, and dysregulation and persistent activation of the alternative complement pathway [64]. On the other hand, the results from in vitro studies indicated that various components of *Perilla frutescens* extracts, and especially rosmarinic acid, have the potential to reduce vascular permeability and leukocyte migration, cytokine and chemokines secretion, specific antibody production, and nitric oxide production [65,66,67]. Thus, the *Perilla frutescens* extracts could be considered a viable candidate as an adjuvant therapeutic agent for the modulation of the local inflammatory response characteristic of mesangioproliferative glomerulonephritis.

Researchers should keep in mind that several reports indicated a potential toxicity associated with exposure to *Perilla frutescens* [68,69], and this aspect is particularly important for those patients with severe forms of IgA in whom the estimated glomerular filtration rate is reduced and who need appropriate dose adjustments to avoid progression to end-stage renal disease and need dialysis.

The toxicity of *Perilla frutescens* extracts was studied in animal models. It was hypothesized that the most toxic component of this plant is Perilla ketone, which induced pulmonary edema in laboratory animals [70]. Mice and hamsters exhibited relatively low intraperitoneal median lethal dose values (5 mg/kg and 13.7 mg/kg, respectively), whereas dogs and pigs required significantly higher lethal doses (106 mg/kg and 158 mg/kg, respectively) [71]. The predominant manifestation of perilla ketone-associated pathology in dogs and pigs was observed in the liver, with minimal impact on the pulmonary tissue. Conversely, mice and hamsters exhibited solely pulmonary lesions in response to Perilla ketone exposure.

A recent study by Zhang et al. investigated the acute and sub-chronic 90-day oral toxicity of *Perilla frutescens* seed oil in rodent and dog models [72]. In the acute oral toxicity study, the authors reported no significant treatment-associated toxicity or mortality using *Perilla frutescens* seed oil dosages of up to 50 g/kg in KM mice and 20 g/kg in Wistar rats; thus, the median lethal dose (LD_50_) could not be calculated. In the sub-chronic stage, the authors reported soft or mucoid feces along with decreased food consumption in dogs that received high doses of *Perilla frutescens* seed oil (12 g/kg/day). The urinalysis of these subjects did not reveal any changes in the evaluated parameters (urine occult blood, nitrites, pH, urobilinogen, bilirubin, proteins, glucose, or ketones).

The results from this meta-analysis should be evaluated considering some inherent limitations. First of all, we could not assess all outcomes presented in the studies due to the limited information extracted, which did not allow a coherent statistical analysis. Secondly, we could not include in this study randomized controlled trials because none were conducted until now, and the results are based on interventional studies on animals.

Further studies on larger cohorts of human subjects or several randomized controlled trials could represent scientific material of higher quality for the next meta-analysis. Meanwhile, we consider that our results support some of the favorable outcomes that result from *Perilla frutescens* administration.

## 4. Materials and Methods

We performed a systematic search of published studies that evaluated the therapeutic effects of *Perilla frutescens* extracts on various models of IgA nephropathy in MEDLINE, EMBASE, and Cochrane Library using synonyms of ‘*Perilla frutescens*’, ‘chronic renal disorder’, ‘nephropathy’, ‘Berger disease’, and ‘glomerulopathy’, and Boolean operators AND/OR, in accordance with Preferred Reporting Items for Systematic Reviews and Meta-Analyses (PRISMA) guidelines (Supplementary Material Table S1: PRISMA 2020 checklist).

Additional research consisted of a manual screening of references cited in the evaluated papers in order to ensure that all relevant studies were included. Duplicates were removed using EndNote software version 20.4 (Clarivate, Philadelphia, PA, USA).

This systematic review and meta-analysis is registered in the Open Science Framework (DOI: https://doi.org/10.17605/OSF.IO/MZXYE) and PROSPERO-International prospective register of systematic reviews (ID: CRD42023389256) registries. The time frame settled for this research was from inception up to 31 of December 2022, with an English language restriction.

The inclusion criteria were represented by interventional studies on HIGA and Wistar rats, with a therapeutic study design that compared the therapeutic effects of at least one *Perilla frutescens* with a control group. We excluded opinion papers, studies that were published in a language other than English, studies in humans or in sillico, and case reports from the search.

The full-text papers were independently reviewed by two investigators (G.A. and A.-M.A.) to establish their eligibility for the review. Any differences between the two were remedied by a third reviewer (M.H.) if a consensus could not be reached. Two investigators (G.A. and V.H.) retrieved data from the eligible studies separately using a standard process. Data concerning the first author, publication year, study design, characteristics of the population examined, number of cases and controls, and therapeutic outcomes were obtained.

The primary outcome evaluated was represented by proteinuria expressed quantitatively (mg/24 ore) or semiquantitatively on a dipstick (proteinuria: − = negative, B = trace; + = 30 mg/dL; ++ = 100 mg/dL, and +++ = 300 mg/mL). The secondary outcomes included the following:

1. Serum IgA levels (mg/dL);
2. Proliferating cell nuclear antigen (PCNA)—the average number of PCNA positive-cells in a glomerular cross section;
3. [^3^H]Thymidine incorporation (cpm).

Two independent reviewers (I.A.V. and A.H.) assessed the methodological quality of the included studies using SYRCLE’s risk of bias tool [73]. Any disagreements were resolved by discussion with a third reviewer (S.R.). For studies that did not provide sufficient information for a meta-analysis, we chose to present their results in a descriptive manner.

We summarized the data from each study and calculated the standardized mean difference for continuous outcomes. The meta-analysis was performed using a random effects model due to the small cohorts of subjects. In order to show variation and to explore the heterogeneity of the results, we drew forest plots, and we calculated the I^2^ statistic for a quantification of the degree of heterogeneity. A funnel plot was employed for the evaluation of publication bias among studies. The statistical analyses were performed using STATA SE (version 17, 2021, StataCorp LLC, College Station, TX, USA).

## 5. Conclusions

This systematic review and meta-analysis indicated that administration of *Perilla frutescens* extracts led to a statistically significant reduction in proteinuria and PCNA levels in rats that received high doses of the extract as well as in the PCNA level and DNA synthesis in rats that received low doses of the extract.

The pathophysiological background of mesangioproliferative glomerulonephritis is defined by an important local inflammatory response, and *Perilla frutescens* could potentially modulate this inflammatory response in order to obtain better clinical outcomes.

## 6. Future Directions of Research

The results of this systematic review and meta-analysis constitute a basis for further clinical trials on animal models and humans that could evaluate the influence of *Perilla frutescens* extracts administration over the histological markers of progression, clinical evolution, and interaction with other therapeutic agents, as our results benefited from a low degree of heterogeneity.

Since the pattern of mesangioproliferative glomerulonephritis is dynamic and can progress to more severe forms, it is important to identify specific clinical, histological, and molecular targets for various therapeutic agents that would modulate the disease’s evolution and progression to end-stage renal disease.

This study outlined several markers that could potentially be targeted by *Perilla frutescens* extracts and indicated overall good results. However, it is important to further evaluate the optimal dosage in humans as these extracts have proven to have a certain degree of toxicity.

## Figures and Tables

**Table 1:** Characteristics of the included studies.

Author	Year	Number of Subjects Included	Animal Model/Cells	Parts Used	Extract	Treatment Groups
Makino et al. [31]	1999	46	HYGA mice	leaves	*Perilla frutescens* var, crispa, Labiatae	LD (50 mg/kg/day) HD (500 mg/kg/day)
Makino et al. [32]	2001	30	Wistar rats	leaves	*Perilla* *frutescens* Britton var. crispa	LD (100 mg/kg/day) HD (500 mg/kg/day)
Makino et al. [33]	2002	24	Wistar rats	leaves	*Perilla frutescens*	Rosmarinic acid (100 mg/kg/day)
Makino et al. [34]	2003	42	HIGA mice	leaves	*Perilla* *frutescens* Britton var. crispa	LD (50 mg/kg/day) HD (500 mg/kg/day)
Sakurai et al. [35]	2011	16	HIGA mice	seed oil	*Perilla frutescens*	Perilla seed oil (70 g/kg)

Table legend: IgA—immunoglobulin A; HIGA—High-IgA-prone congenic mice; LD—low-dose group; HD—high-dose group.

**Table 2:** Quality assessment of the included studies using SYRCLE’s risk of bias tool for animal studies.

Study	Selection Bias			Performance Bias		Detection Bias		Attrition Bias	Reporting Bias	Other
	Was the Allocation Sequence Adequately Generated and Applied?	Were the Groups Similar at Baseline or Were They Adjusted for Confounders in the Analysis?	Was the Allocation Adequately Concealed?	Were the Animals Randomly Housed during the Experiment?	Were the Caregivers and/or Investigators Blinded from Knowledge of Which Intervention Each Animal Received during the Experiment?	Were Animals Selected at Random for Outcome Assessment?	Was the Outcome Assessor Blinded?	Were Incomplete Outcome Data Adequately Addressed?	Are Reports of the Study Free of Selective Outcome Reporting?	Was the Study Apparently Free of Other Problems that could Result in High Risk of Bias?
Makino et al. [31]	Unclear	Yes	Unclear	Unclear	No	No	Unclear	Yes	Yes	Yes
Makino et al. [32]	Unclear	Yes	Unclear	Unclear	No	No	Unclear	Yes	Yes	Yes
Makino et al. [33]	Unclear	Yes	Unclear	Unclear	No	No	Unclear	Yes	Yes	Yes
Makino et al. [34]	Unclear	Yes	Unclear	Unclear	No	No	Unclear	Yes	Yes	Yes
Sakurai et al. [35]	Yes	Yes	Unclear	Unclear	Unclear	No	Unclear	Yes	Yes	Yes

**Table 3:** The results of the random effects model used for meta-analysis.

Outcome	Groups	Effect Size	Std. Error	Z	Sig. (Two-Tailed)	Lower Limit 95% CI	Upper Limit 95% CI
Proteinuria	LD vs. placebo	−0.85	0.866	−1.040	0.30	−2.45	0.75
	HD vs. placebo	−1.91	0.499	−4.081	<0.001	−2.91	−0.91
IgA	LD vs. placebo	−2.36	3.795	−0.977	0.32	−6.99	2.28
	HD vs. placebo	−2.44	2.085	−1.214	0.22	−6.37	1.49
PCNA	LD vs. placebo	−2.94	0.653	−4.856	<0.001	−4.06	−1.82
	HD vs. placebo	−2.86	0.861	−3.320	<0.001	−4.54	−1.17
[^3^H] thymodine	LD vs. placebo	−1.60	0.627	−2.682	<0.001	−2.68	−0.52
	HD vs. placebo	−2.39	1.731	−1.460	0.14	−5.46	0.68

Table legend: IgA—immunoglobulin A; PCNA— proliferating cell nuclear antigen; [^3^H] thymidine incorporation; CI—confidence interval.

## Data Availability

This systematic review and meta-analysis is registered in the Open Science Framework Registry (DOI: https://doi.org/10.17605/OSF.IO/MZXYE), and it is available on: https://archive.org/details/osf-registrations-mzxye-v1 (Accessed on 20 May 2023). This study is also registered in PROSPERO—International prospective register of systematic reviews (ID: CRD42023389256), available on: https://www.crd.york.ac.uk/PROSPERO/#myprospero (accessed on 21 May 2023). Additional data are available upon a reasonable request from the corresponding author due to local policies.

## Supplementary Materials

The following supporting information can be downloaded at: https://www.mdpi.com/article/10.3390/ph16070988/s1, Table S1: PRSIMA 2020 checklist.
